# Remote care and triage of obstetric patients with COVID-19 in the community: operational considerations

**DOI:** 10.1186/s12884-022-04863-0

**Published:** 2022-07-08

**Authors:** Charles Bircher, Matt Wilkes, Nicole Zahradka, Emily Wells, Ed Prosser-Snelling

**Affiliations:** 1 Maternity Department, Norfolk and Norwich University Hospital, Colney Ln, Norwich, NR4 7UY England; 2 Clinical Research, Current Health Ltd, Playfair House, 6 Broughton St Ln, Edinburgh, EH1 3LY Scotland

**Keywords:** Telemedicine, Remote consultation, Pregnancy, Change management, Delivery of healthcare

## Abstract

**Background:**

During the SARS-CoV-2 (COVID-19) pandemic, routine antenatal care was disrupted, and pregnant women positive for COVID-19 were at increased risk of caesarean section, intensive care admission or neonatal unit admission for their baby. Virtual care and telehealth can reduce barriers to care and improve maternity outcomes, and adoption has been encouraged by health authorities in the United Kingdom.

**Methods:**

Norfolk and Norwich University Hospitals Trust deployed a flexible maternity virtual ward (MVW) service using the Current Health platform to care for pregnant women during the pandemic. Patients were monitored either intermittently with finger pulse oximetry or continuously with a wearable device. We outline the MVW technology, intervention and staffing model, triage criteria and patient feedback, as an example of an operational model for other institutions.

**Results:**

Between October 2021 and February 2022, 429 patients were referred, of which 228 were admitted to the MVW. Total bed-days was 1,182, mean length of stay was 6 days (SD 2.3, range 1–14 days). Fifteen (6.6%) required hospital admission and one (0.4%) critical care. There were no deaths. Feedback alluded to feelings of increased safety, comfort, and ease with the technology.

**Conclusions:**

The MVW offered a safety net to pregnant women positive for COVID-19. It provided reassurance for staff, while relieving pressures on infrastructure. When setting up similar services in future, attention should be given to identifying clinical champions, triage criteria, technology and alarm selection, and establishing flexible escalation pathways that can adapt to changing patterns of disease.

## Background

Pregnant women hospitalised with SARS-CoV-2 (COVID-19) have been more likely to be admitted to critical care, and to require caesarean section or neonatal unit admission for their baby [1–3]. A disproportionate number of those admitted to critical care have been from Black, Asian or Other Minority Ethnic (BAME) groups, overweight, obese, or had another relevant comorbidity [1, 2]. Local Maternity Services have been asked to increase support for at-risk pregnant women, including BAME women [4].

Virtual care and telehealth have been shown to improve outcomes in certain areas of maternal-foetal medicine and have been suggested as a means of breaking down barriers to access in prenatal care during COVID-19 [5–7]. The National Clinical Director for Maternity and Women’s Health and the Chief Midwifery Officer for the U.K. have recommended home oximetry for pregnant women positive for COVID-19 [8]. However, there have been few published examples of how this is accomplished in practice. A key challenge is the identification of sentinel events which predict deteriorations in clinical conditions. The number needed to treat is high: in the UKOSS cohort, the estimated incidence of hospitalisation with symptomatic SARS-CoV-2 was 2.0 per 1000 maternities (95% CI 1.9–2.2) [1]. Each new COVID-19 variant brings new patterns of transmission, virulence and vaccine evasion, which alter national guidance and population behaviours. In turn, these change the frequency of sentinel events and the challenge for monitoring programmes. Maternity services need to continuously improve their programmes of support to hit this moving target. With variants of relatively high transmissibility but low virulence (such as Omicron), numbers of positive patients rapidly increase, and triage of virtual ward admissions become essential to avoid overwhelming capacity.

Norfolk and Norwich University Hospitals Trust navigated these challenges, by deploying a flexible Virtual Ward service to care for vulnerable populations during the pandemic. A virtual ward is designed to provide patients with a period of intensive multidisciplinary management and monitoring, akin to an inpatient stay. Pre-pandemic, virtual wards had been shown to reduce mortality in heart failure, and they were widely deployed in COVID-19 at the behest of NHS England [9, 10]. A recent review found evidence for the effectiveness of virtual wards deploying pulse oximetry alone to be limited, though only one study of maternity patients was included in the analysis [11]. Indeed, while current clinical practice guidelines for management of COVID-19 in pregnancy vary, there remains general consensus that women should be monitored at home if possible, and hospitalised only in the case of respiratory distress, elevated respiratory rate or low oxygen saturation [12]. A need for development of shared guidelines has also been identified [12].

At first the Maternity Virtual Ward (MVW) was offered to all pregnant women with confirmed COVID-19. As volumes increased, a system of triage was developed to cope with demand. We outline the Virtual Ward technology, intervention and staffing model, readmission rates, as well as the specific triage criteria and alarm settings used, as an example of an operational model for other institutions and as a contribution to the emerging consensus around best care [12].

## Methods

### Monitoring platform

The MVW coordinated care through the Current Health platform (Current Health Ltd, Edinburgh, UK). The Current Health platform was a cloud-based analytics system with a web dashboard for the monitoring teams to view the patients’ vital signs and survey responses in real time. The web dashboard displayed the patients’ observations in a format akin to the familiar hospital observation chart. Alarms were set (Table 1) to alert the team via push notification of any deterioration. Vital signs were gathered either intermittently via finger pulse oximetry (AM801 pulse oximeter, Med Linket, Shenzhen, China) or continuously using a Current Health wearable. The wearable collected continuous, clinical-grade measures of oxygen saturation (SpO_2_), respiratory rate, pulse, motion, and skin temperature, and could integrate with a blood pressure cuff, axillary temperature patch and a spirometer. The kit connected to the Current Health cloud via a home internet connection, or a 3G network sim card for those without home internet.

**Table 1:** Alarm settings for continuously monitored patients in the virtual ward

Monitor	Alarm	Setting
AM801 Pulse Oximeter	Hypoxia	SpO2 < = 93
Current Health Wearable	Hypoxia / Tachypnoea	SpO2 < = 90 AND RR > = 25 for 60 min
	Hypoxia / Bradypnoea	SpO2 < = 90 AND RR < = 10 for 60 min
	Tachycardia / Tachypnoea	HR > = 90 and RR > = 25 for 60 min
	Bradycardia	HR < = 45

*HR* heart rate, *RR* respiratory rate, *SpO2* peripheral oxygen saturation

### Inclusion criteria and initial assessment

The MVW identified pregnant patients with confirmed-positive COVID-19 via three routes: discharge from hospital, direct contact from a patient in the community, and positive swabs in the community (Pillar 2 of the National Testing Strategy). Details of those with positive swabs were supplied via a dataset from NHS England, and cross referenced with the maternity database (E3, Wellbeing Software, Mansfield, U.K.). Initially, all women were called by a member of the obstetric medical team to perform a risk assessment for complications from COVID-19 in pregnancy. As the pandemic progressed and numbers grew, midwives were trained to do these initial risk assessments, and the obstetric team only contacted the patients if there were concerns from the midwifery team. All patients continued in the MVW initially, but subsequently only patients meeting any of triage criteria were admitted, to cope with increasing case numbers and target those who would derive most benefit. The triage criteria included ethnicity, age, BMI, comorbidities, vaccination status and socioeconomic deprivation and social support (Table 2). They were taken from the RCOG Coronavirus (COVID-19) Infection in Pregnancy guideline, with the additional risk factor of limited spoken English, to reflect the additional needs of marginalised populations [3]. Patients who did not require hospitalisation, or who did not meet any of the MVW criteria were given isolation advice and signposted to further help should they require it.

**Table 2:** Criteria for Maternity Virtual Ward admission and ongoing risk assessment

Criteria
Women from Black Asian Minority Ethnic Background
Increased maternal age ≥ 35 years
Raised BMI (≥ 25)
Pre-existing comorbidity (diabetes, hypertension, asthma, COPD or other respiratory)
Unvaccinated (or vaccinated > 6 months previously, without booster)
Living in areas or households of increased socioeconomic deprivation
Lack of English, lack of social support, or limited understanding of how to access help

### Monitoring and escalation

Once referred to the MVW, patients were called by a midwife every 12–48 h depending on their level of risk. Their vital signs were monitored either intermittently with the oxygen saturation probe or continuously with the Current Health wearable, depending on the midwife’s judgment of their baseline risk, symptoms, and clinical trajectory. Out of hours monitoring was shared between the obstetric and MVW teams, and at peak there were five midwives assigned to the service.

If alarms were triggered, or there were obstetric or other concerns, patients were contacted then brought into hospital for obstetric or respiratory medical review if necessary. If patients were uncontactable, then the MVW team contacted their next of kin or escalated to a community midwife for a home visit. Patients were discharged after either 10 days in the virtual ward, 10 days from a positive test, or seven days from a positive test with negative lateral flow tests on days six and seven. Consideration was given to thromboprophylaxis at each stage. Growth scans were arranged 14 days post-Covid-19 detection for women who were severely or critically unwell.

### Data collection

Data were collated from the Current Health platform and the hospital electronic medical record (E3, Wellbeing Software, Mansfield, U.K.) and imported into R (R Foundation for Statistical Computing, Vienna, Austria). They included: age, admission dates and length of stay, clinical escalation rates (hospitalisation, critical care, death) and patient feedback. Patient feedback was captured by the NNUH administrative support service after the patient had been discharged from the MVW as service evaluation. Patients were asked to rate the service from 0 (least/worst) to 5 (most/best) in the aspects listed in Table 3. Any additional free text comments were iteratively coded and analysed thematically [13] Quantitative results were assessed for normality (visualisation, Shapiro–Wilk test), and presented as mean (SD).

**Table 3:** Responses to the Maternity Virtual Ward Patient Survey. (*n* = 24). Patients were asked to score the service from 0 (“least”/ “worst”) to 5 (“most”/ “best”)

Question		
	Mean Score	Range of scores
Do you feel you were given all the information you needed before being transferred onto the Virtual Ward?	5.0	5
How easy do you feel it was to use the technology?	5.0	5
Did being part of the Virtual Ward make you feel more confident in leaving hospital?	5.0	4–5
Would you use the service again? And would you recommend to family and friends?	4.9	4–5
Overall how do you feel about the service you received from the NNUH Virtual Ward Team?	5.0	4–5

## Results

### Referrals and admission

**Fig. 1:**
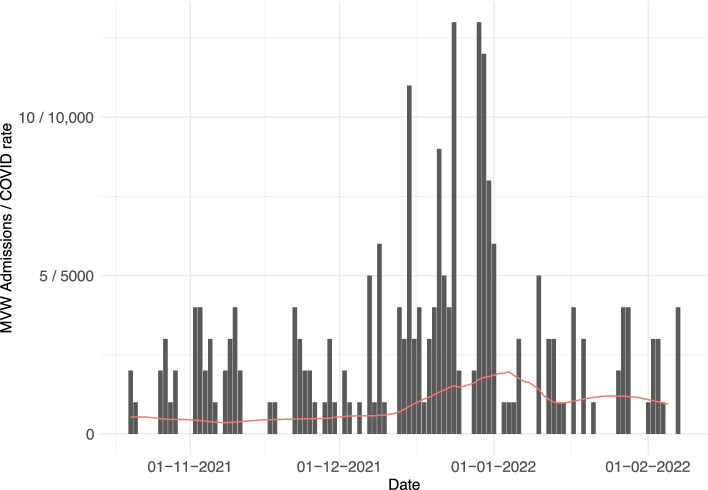
Admissions to the Maternity Virtual Ward, presented alongside rolling 7-day rate of new cases of COVID-19 per 100,000 people in the East of England region (data from https://coronavirus.data.gov.uk/)

Between the 20 October 2021 and 7 Feb 2022, 429 patients were referred to the MVW. Following triage, 228 were admitted (Fig. 1), with a mean age of 30.6 (SD 5.6, range 16–44), and all stages of gestation.

### Length of stay and escalation

Total bed-days on the MVW was 1,182 days, with mean length of stay of 6 days (SD 2.3, range 1–14 days). Fifteen (6.6%) required escalation to hospital care, and one (0.4%) to critical care. There were no deaths.

### Feedback

The results of the feedback survey (*n* = 24) are presented in Table 3. Maximal, or near maximal scores were given for patient information, confidence, ease of use, confidence, and overall service. Free text comments alluded to feelings of increased safety, comfort, and ease with the technology.

## Discussion

The MVW brought benefits for patients, healthcare professionals, and the hospital system. It offered monitoring and reassurance for pregnant women positive for COVID-19, peace of mind for obstetricians, with a mean length of stay shorter than the national average reported in home oximetry for COVID-19 (6.0 vs. 12.7 days) and an escalation rate to inpatient care of 6.6% [11]. However, as the pandemic disrupted the normal schedule of antenatal care in the UK, it was also a route to antenatal services for women who were self-isolating, vulnerable, or otherwise struggling to access care. It brought a degree of continuity known to improve satisfaction, and reduce intervention rates [14]. As a safety net, it allayed anxiety for patients and providers alike, and offered a ‘third option’ between primary care and admission, that helped ease pressure on hospital infrastructure and general practice. The technological aspects of the virtual ward performed well, and staff judged the triage criteria and alarm settings to have had the right balance of sensitivity and specificity.

The key challenge was digital transformation. The initial set up and coordination of the MVW required dedication, and a degree of “internal marketing” from enthusiastic individuals to bring the rest of team onboard. The key barrier to engagement was a lack of perceived importance of remote monitoring. Maternity services, especially during COVID-19, did not sit in isolation, so care pathways also had to be coordinated with respiratory, acute and general medicine. Healthcare professionals beyond the MVW team needed to understand that any temporary adjustments to their workflow would be rapidly offset by a reduction in demands on their time once the service had shouldered the load.

The MVW also relied on a core group of midwives skilled in telephone triage and emotional support. Telephone triage is generally considered safe, though risks increase in step with patient acuity and triage protocols are key to its safe implementation [15]. Even with clear admission criteria and escalation pathways, the midwives needed experience and confidence to make composite judgments that integrated the results of the monitoring, the patients’ clinical trajectories and the services available. Midwives were not extensively trained in this, and they had to balance expectations of ‘usual care’ with the capacity of the hospital during the exceptional circumstances of the pandemic.

Clinical leadership is essential for driving this kind of digital transformation [16]. The pandemic created an overwhelming sense of urgency but building a coalition for change starts with strong and credible clinical leaders. Clinical leaders should then build out a team of trained individuals responsible for the execution of the programme. In the NNUH programme, a strong team ethos was essential to maintaining morale, even when working remotely. When working remotely, staff should also have access to the usual services of the hospital (for example, arranging ultrasound scans), so they are not limited in the care that they can offer.

In their evaluation of NHS Virtual Ward programs, Alboksmaty et al. noted the importance of adequate infrastructure and human resources to staff the program, patient education, and appropriate alarm thresholds, alongside the need to report escalation rates [11]. We would build on this by recommending that clinical pathways should include triage criteria, triggers for escalation, pre-agreed admitting locations, and allocation of responsibility for patients at each stage. Pathways must equally include a degree of flexibility, and a process for rapid evaluation and change control, so they can adapt to a rapidly moving situation, though we recognise the inherent tension between adaptability and ensuring consistency of care [16]. The pathways, and the virtual ward service should be ‘marketed’ within the institution, so those peripherally involved are aware of its availability, capability, and potential benefits.

Technology should be chosen that can monitor the desired parameters accurately using validated, CE-marked sensors. Regarding pulse oximeters specifically, clinicians should be aware of the potential for overestimation of SpO_2_ in hypoxic patients with darker skin, and the differences between commercial and clinical-grade pulse oximeters [17, 18]. Facilities for video calling, simultaneous translation or cellular (as well as WiFi) connection may be essential, particularly in areas of social deprivation. A solution that is easily integrated with existing workflows and maternity systems, and that can maintain patient confidentiality while facilitating clinical handover is also desirable. Alarms should be set to balance sensitivity with specificity, as false alarms can be more laborious and disruptive to resolve when the patient is remote. Reported approaches have included fixed thresholds for resting (typically 92%) and post-exertional (> 3–5% decrease) SpO_2 _[11]. In the MVW alarm settings, a time window of 60 min, and combination alarms from multiple vital sign parameters were added to improve specificity in continuous monitoring alarms, to ensure that any alerts reflected the patient’s true physiological state and not a temporary derangement from activities of daily living. Attention should be given to how patients will be contacted if they cease transmitting data, and involvement of the community midwifery service at an early stage is helpful.

## Conclusions

The Virtual Maternity Ward offered (and continues to offer) a safety net to pregnant women who were positive for COVID-19, and those who were struggling to access care. It provided reassurance for staff, while relieving pressures on infrastructure. When setting up similar services in future, attention should be given to identifying clinical champions, triage criteria, and technology selection, and establishing flexible pathways.

## Data Availability

The datasets analysed during the current study are not publicly available due to information governance requirements at NNUH, however they are available from the corresponding author on reasonable request.
